# *Neisseria gonorrhoeae* evades autophagic killing by downregulating CD46-cyt1 and remodeling lysosomes

**DOI:** 10.1371/journal.ppat.1007495

**Published:** 2019-02-12

**Authors:** Won J. Kim, Annette Mai, Nathan J. Weyand, Maria A. Rendón, Koenraad Van Doorslaer, Magdalene So

**Affiliations:** 1 BIO5 Institute, University of Arizona, Tucson, AZ, United States of America; 2 Department of Immunobiology, University of Arizona, Tucson, AZ, United States of America; 3 Department of Biological Sciences, Ohio University, Athens, OH, United States of America; 4 School of Animal and Comparative Biomedical Sciences, University of Arizona, Tucson, AZ, United States of America; University of Illinois, UNITED STATES

## Abstract

The Gram-negative human pathogen *N*. *gonorrhoeae* (Ngo) quickly attaches to epithelial cells, and large numbers of the bacteria remain on the cell surface for prolonged periods. Ngo invades cells but few viable intracellular bacteria are recovered until later stages of infection, leading to the assumption that Ngo is a weak invader. On the cell surface, Ngo quickly recruits CD46-cyt1 to the epithelial cell cortex directly beneath the bacteria and causes its cleavage by metalloproteinases and Presenilin/γSecretease; how these interactions affect the Ngo lifecycle is unknown. Here, we show Ngo induces an autophagic response in the epithelial cell through CD46-cyt1/GOPC, and this response kills early invaders. Throughout infection, the pathogen slowly downregulates CD46-cyt1 and remodeling of lysosomes, another key autophagy component, and these activities ultimately promote intracellular survival. We present a model on the dynamics of Ngo infection and describe how this dual interference with the autophagic pathway allows late invaders to survive within the cell.

## Author summary

*Neisseria gonorrhoeae* (Ngo), which causes the sexually transmitted disease of gonorrhea, primarily infects the uorgenital epithelium. It attaches to the epithelial surface for lengthy periods. It also invades epithelial cells, but few viable intracellular bacteria are recovered until later stages of infection. As Ngo is known to interfere with two key components in the autophagic pathway, we determined the influence of this host defense mechanism on the lifecycle of the pathogen. We report that Ngo induces autophagy in human primary cervical epithelial cells as well as endorvical cell lines ME180 and Hec1B. Autophagy is induced through the CD46-cyt1/GOPC pathway and this response kills Ngo invading cells early in infection. Throughout infection, Ngo mounts a counter-attack on the autophagic pathway by downregulating CD46-cyt1 and disturbing lysosome homeostasis. This interference allows late-invading Ngo to escape autophagic killing.

## Introduction

Autophagy is critical for cellular homeostasis [1]. Highly conserved from yeast to man, this catabolic process sequesters aging or damaged cytoplasmic contents and organelles in a structure called the autophagosome [2–4]. The autophagosome then fuses with the lysosome to form the autophagolysosome, where lysosomal enzymes degrade the sequestered contents for recycling [2–4]. Cells starved for nutrients also upregulate autophagy to hasten the recycling of their cytoplasmic contents [2, 5].

Studies in yeast have elucidated many steps in autophagy. A double membrane structure called the isolation membrane forms around the cargo targeted for degradation [2, 3]. Initiation of this step requires the class III P(I) kinase VPS34 and Beclin1 [3, 6]. The isolation membrane then elongates and its ends fuse, forming a vesicle called the autophagosome [4]. During isolation membrane elongation, the cytosolic protein LC3-I is conjugated to phosphatidylethanolamine, and the resulting lipidated product, LC3-II, is incorporated into the inner and outer membranes of the autophagosome [3, 7, 8]. LC3-II plays an important role in cargo selection through binding to adaptor molecules that are associated with damaged cytoplasmic contents [7, 8].

Eukaryotic cells also mount an autophagic response called xenophagy against intracellular pathogens [9]. Several pathways that target intracellular bacteria and viruses for degradation have been described. Autophagic destruction of *Salmonella typhimurium* is initiated by the binding of Galectin-8 to damaged *Salmonella* containing vacuoles (SCVs) [10]. Autophagy receptor NDP52 binds both Galectin-8 and LC3-II, and targets SCVs for autophagic degradation [10, 11]. In a similar manner, receptors optineurin and p62 recruit the autophagic machinery to the site of intracellular pathogens [10–13]. Autophagic destruction of Group A *Streptococcus* (GAS) and measles virus is initiated by CD46-cyt1, an isoform of the ubiquitously expressed Type I membrane protein CD46. Upon CD46-cyt1 engagement, its cytoplasmic tail interacts with scaffold protein GOPC, thereby recruiting the VPS34/Beclin-1 complex that initiates autophagy [14].

The Gram-negative sexually transmitted pathogen *Neisseria gonorrhoeae* (Ngo) interacts with CD46-cyt1 at several levels [15–18]. Ngo uses the Type IV pilus (Tfp) and opacity-associated proteins (Opa) to attach to epithelial cells, and remains on the cell surface for prolonged periods without causing damage [19–24]. Via its Tfp, Ngo quickly recruits CD46-cyt1 to the site of infection [16]. Ngo stimulates matrix metalloproteinases to cleave the CD46-cyt1 ectodomain, causing its shedding, and the Presenilin/γSecretase complex to cleave its transmembrane domain, causing its release [17]. CD46-cyt1 downregulation occurs slowly, but by 9 hours post-infection (hpi), total cellular levels of CD46-cyt1 are significantly reduced in infected cells [15, 17, 18]. The importance of Ngo-CD46-cyt1 interactions to the Ngo lifecycle is unknown.

The involvement of CD46-cyt1 in the autophagic response to GAS and measles infection led us to test the hypothesis that Ngo engagement of CD46-cyt1 stimulates autophagy [14]. In our study, we used Ngo MS11, a piliated and Opa-nonexpressing strain, as Ngo interacts with CD46-cyt1 through Tfp, not Opa. Using high-resolution microscopy, immunoblots, siRNA knockdowns and chemical inhibitors, we show that Ngo induces autophagy through the CD46-cyt1/GOPC pathway. This response kills intracellular Ngo early in infection. However, there is an increase in intracellular viable counts at later time points. We show that this increase is due to pathogen downregulation of CD46-cyt1 and perturbation of lysosome homeostasis. We discuss our findings in the context of Ngo intracellular survival strategies, and provide a model to explain how Ngo interferes with autophagic flux over the course of infection to promote its eventual intracellular survival within the host cell.

## Results

### Ngo infection induces autophagosome formation

**Fig 1:**
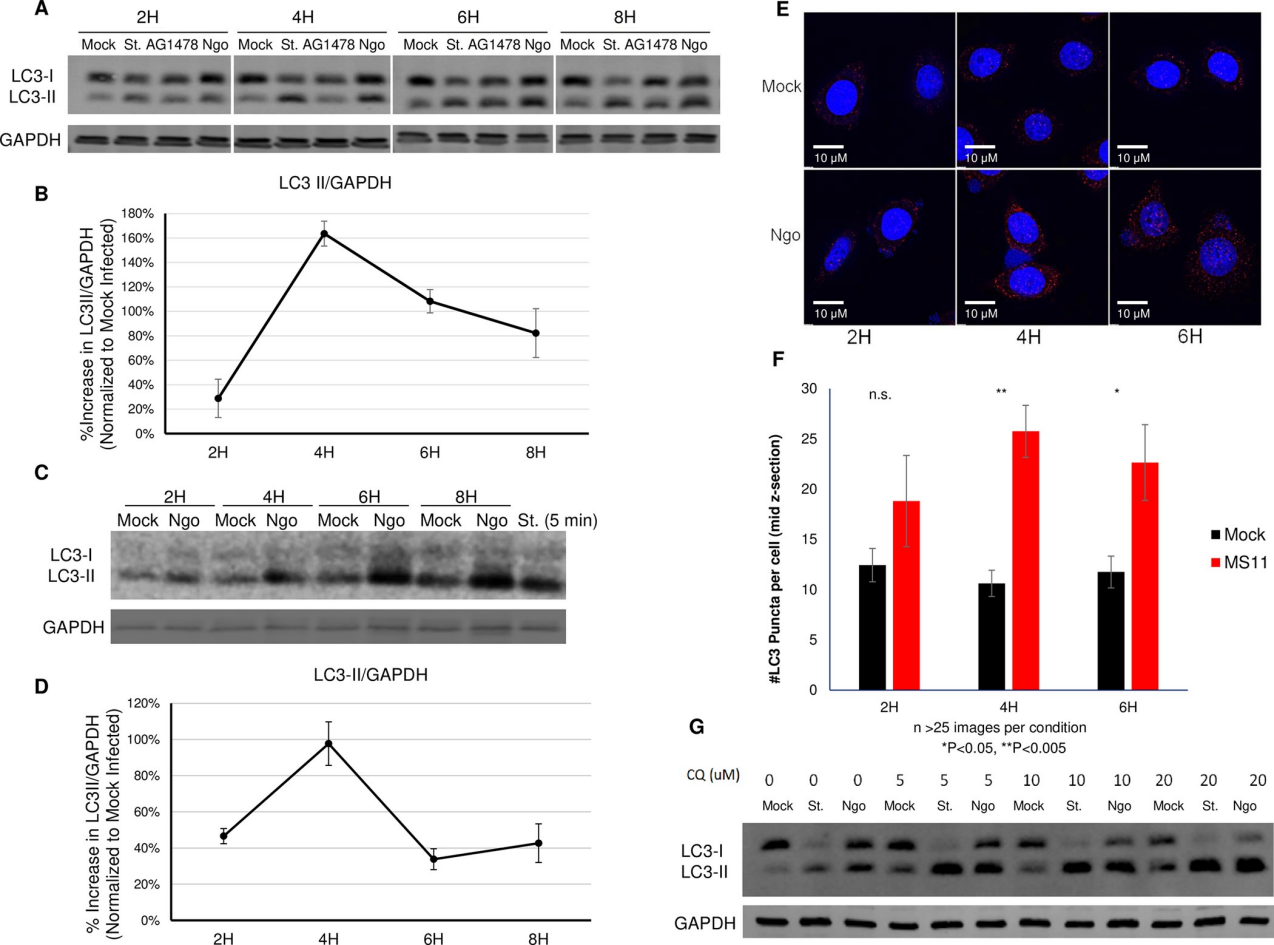
Ngo infection induces autophagic flux. (A)Representative immunoblot showing LC3-I, LC3-II and GAPDH in ME180 cells that were mock infected, starved (St.) for 4 h, treated with EGFR inhibitor AG1478, or infected with Ngo strain MS11 at an MOI of 10 for 2, 4, 6 or 8 h. GAPDH served as the internal control for each sample. (B)Densitometry quantification of LC3-II levels in 5 independent immunoblots (described in A). LC3-II levels in each lane were normalized to the internal GAPDH signal, and the normalized values were expressed relative to that in mock-infected cells. Error bars represent standard error of the mean (SEM). Statistical analysis was performed using student’s *t-*test. (C)Representative immunoblot showing LC3-I, LC3-II and GAPDH in primary human endocervical epithelial cells that were mock infected, starved (St.) for 5 minutes, or infected with Ngo at an MOI of 10 for 2, 4, 6, or 8 h. GAPDH served as the internal control for each sample. (D)Densitometry quantification of LC3-II levels in 3 independent immunoblots (described in C). LC3-II levels in each lane were normalized to the internal GAPDH signal, and the normalized values were expressed relative to of that in mock-infected cells. Error bars represent standard error of the mean (SEM). Statistical analysis was performed using student’s *t-*test. (E)Representative deconvolution images of ME180 cells either mock or Ngo infected for 2, 4, or 6 h. Samples were stained with DAPI (blue) and LC3 antibody (red). (F)Quantification of LC3-positive puncta in ME180 cells treated as described in C, from 3 independent experiments. In each experiment, >25 images per condition were analyzed. The mid z-section of each image was analyzed for the number of LC3+ puncta using ImageJ software Analyze Particle function. Error bars represent SEM. Statistical analysis was performed using student’s *t-*test (G)Representative immunoblot (n = 2) showing LC3-I, LC3-II and GAPDH in ME180 cells that were treated with 0, 5, 10, or 20 μM of CQ for 30 min, then mock infected, starved (St.), or infected with Ngo at an MOI of 10.

We determined whether MS11, a piliated, Opa-nonexpressing Ngo strain, induces autophagic flux in the human endocervical epithelial cell line ME180, using immunoblotting to monitor the level of the autophagosome marker LC3-II. As expected, starvation (st), the positive control [2, 5], quickly induced the accumulation of LC3-II (Fig 1A). EGFR kinase inhibitor AG1478, a second positive control, also induced LC3-II accumulation by 6 h post-treatment [25, 26]. Ngo infected cells also had higher levels of LC3-II compared to mock-infected cells (Fig 1A and 1B). This increase was detected as early as 2 hours post-infection (hpi), peaked at 4 hpi, and gradually decreased thereafter. Similarly, Ngo induced the accumulation of LC3-II in human primary cervical epithelial cells (Fig 1C and 1D). Normalized LC3-II levels also peaked at 4 hpi and decreased at 6–8 hpi.

To determine whether Ngo induces the formation of autophagosomes, we quantitated LC3-positive cellular structures using deconvolution microscopy. Mock-infected ME180 cells had few LC3+ puncta; this state represents autophagosomes at the basal level (Fig 1E and 1F). Infected cells had significantly higher numbers of LC3+ puncta than mock-infected cells (Fig 1E and 1F). These results suggest that autophagy is induced upon Ngo infection.

Upon fusion of the lysosome with the autophagosome, lysosomal enzymes degrade LC3-II in the inner membrane of the autophagosome, along with autophagic contents. Inhibiting this final step in autophagy leads to the accumulation of LC3-II [7, 8]. The higher levels of LC3-II in Ngo-infected cells could be caused by the induction of autophagy or inhibition of degradation of molecules in the autophagosome [7, 8, 27]. To test whether the increased LC3-II level is mediated by lysosomal inhibition, we blocked LC3-II degradation using the lysosome inhibitor chloroquine diphosphate (CQ). This allowed us to measure the accumulation of LC3-I and its conversion to LC3-II over time. In CQ-treated ME180 cells, LC3-II accumulated at higher levels in infected cells than mock-infected cells (Fig 1G, S1 Fig). While we cannot rule out the possibility that degradation of autophagic cargos is also inhibited, these results strongly suggest that Ngo infection hastens the conversion of LC3-I to LC3-II.

LC3-mediated phagocytosis (LAP), a process similar to autophagy, has been characterized in dendritic cells and bone marrow derived macrophages (BMDM) [28, 29]. In contrast to autophagy, LAP requires Rubicon to activate the class III P(I) kinase [28, 29]. We attempted to determine whether LAP plays a role in LC3-II accumulation during infection. Rubicon levels in epithelial cells were undetectable compared to that in Bone Marrow Derived Macrophages (BMDMs) (S2 Fig), strongly suggesting that Ngo infection induces autophagy rather than LAP.

### Intracellular Ngo are found in autophagosomes and autophagolysosomes

**Fig 2:**
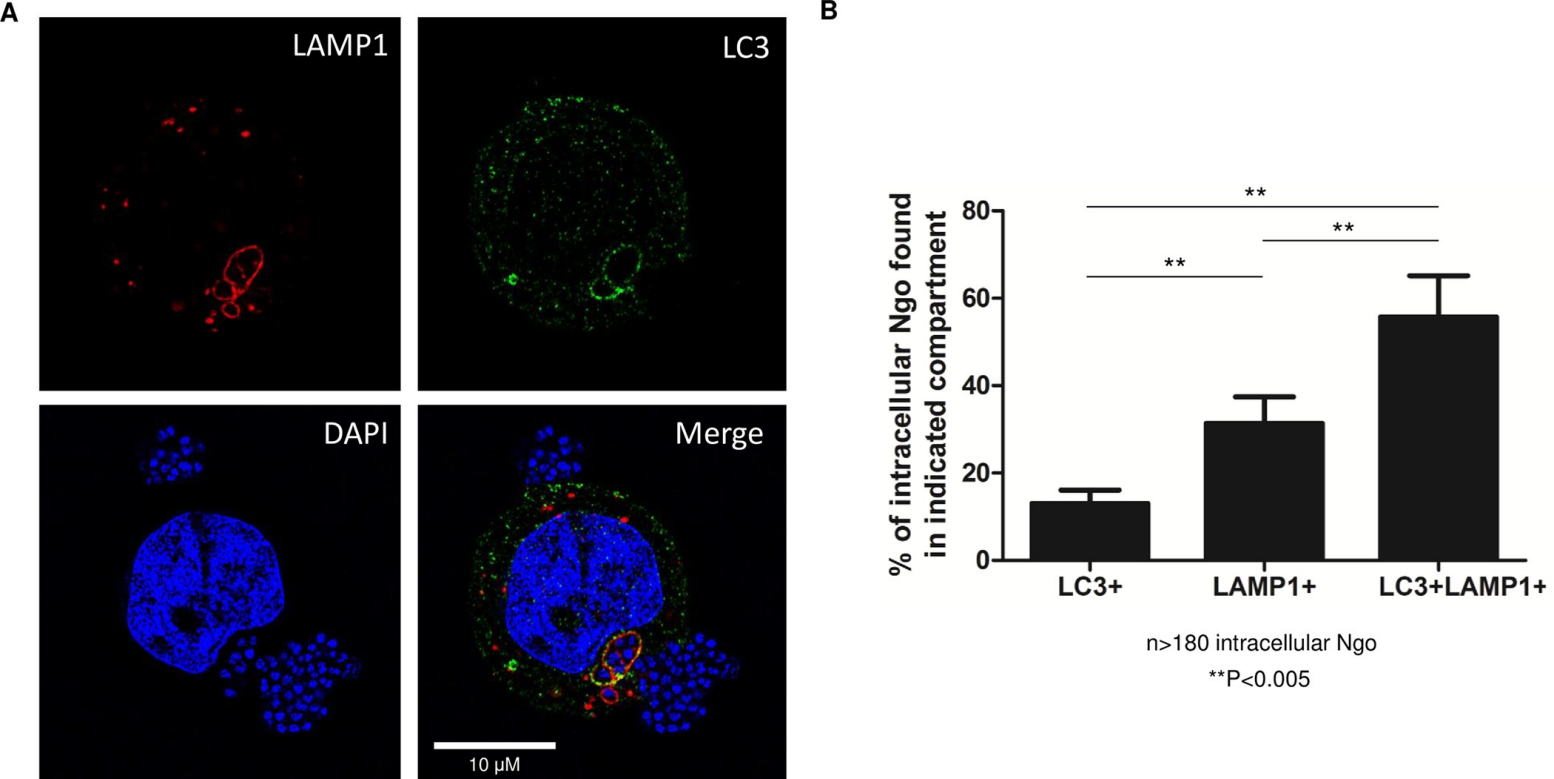
Intracellular Ngo are in autophagosomes and autophagolysosomes. (A)Representative Super Resolution (SIM) microscopy image of an ME180 cell treated with Ctrl shRNA, infected with Ngo for 4 h at an MOI of 10, and stained for LAMP1 (red), LC3 (green), and DAPI (blue). The small clusters of DAPI signal indicate Ngo. Arrowhead in the lower right panel (Merge) shows intracellular Ngo in a LC3+LAMP1+ compartment. (B)Quantitation of intracellular Ngo in SIM images as described in (A). Randomly selected fields of ME180 cells were selected for analysis. For each imaged cell, the number of intracellular Ngo (punctate blue DAPI signal) surrounded by LC3, LAMP1+, or LC3+ LAMP1+ signals were counted. 3 independent experiments were performed. 25 epithelial cells were analyzed (185 intracellular bacteria). Statistical analysis was performed using student’s *t-*test.

We used Structured Illumination Microscopy (SIM) to determine whether intracellular Ngo is targeted to autophagosomes. ME180s cells were infected with Ngo for 4 h, stained with LC3, LAMP1 (endosome/lysosome marker), and DAPI (DNA marker), and examined by SIM. Small clusters of Ngo (blue due to DAPI staining) colocalized with LC3, LAMP1 or LC3/LAMP1 signals (Fig 2A). The colocalization with LC3/LAMP1 was consistent throughout the length of the infected cells (S3 Fig). In 3D constructed Z-section images, the LC3, LAMP1 and LC3/LAMP1 signals surrounding Ngo resembled spherical/ellipsoidal compartments (S1A and S1B Video), suggesting that intracellular Ngo are located in autophagosomes (LC3+), endosomes/lysosomes (LAMP1+), or autophagolysosomes (LC3+, LAMP1+).

The same SIM images were examined for the prevalence of Ngo in lysosomes/late endosomes (LAMP1+), autophagosomes (LC3+), and autophagolysosomes (LC3+, LAMP1+). Results show 31% of Ngo were in lysosomes/late endosomes, and 13% in autophagosomes (Fig 2B). The majority (56%) occupied autophagolysosomes (Fig 2B). These data lend further support to the previous observation that Ngo induces the formation of autophagosomes and autophagolysosomes. Furthermore, they suggest that at 4 hpi, intracellular Ngo were predominantly located in autophagolysosomes.

### Ngo infection induces the CD46-cyt1/GOPC autophagy pathway

**Fig 3:**
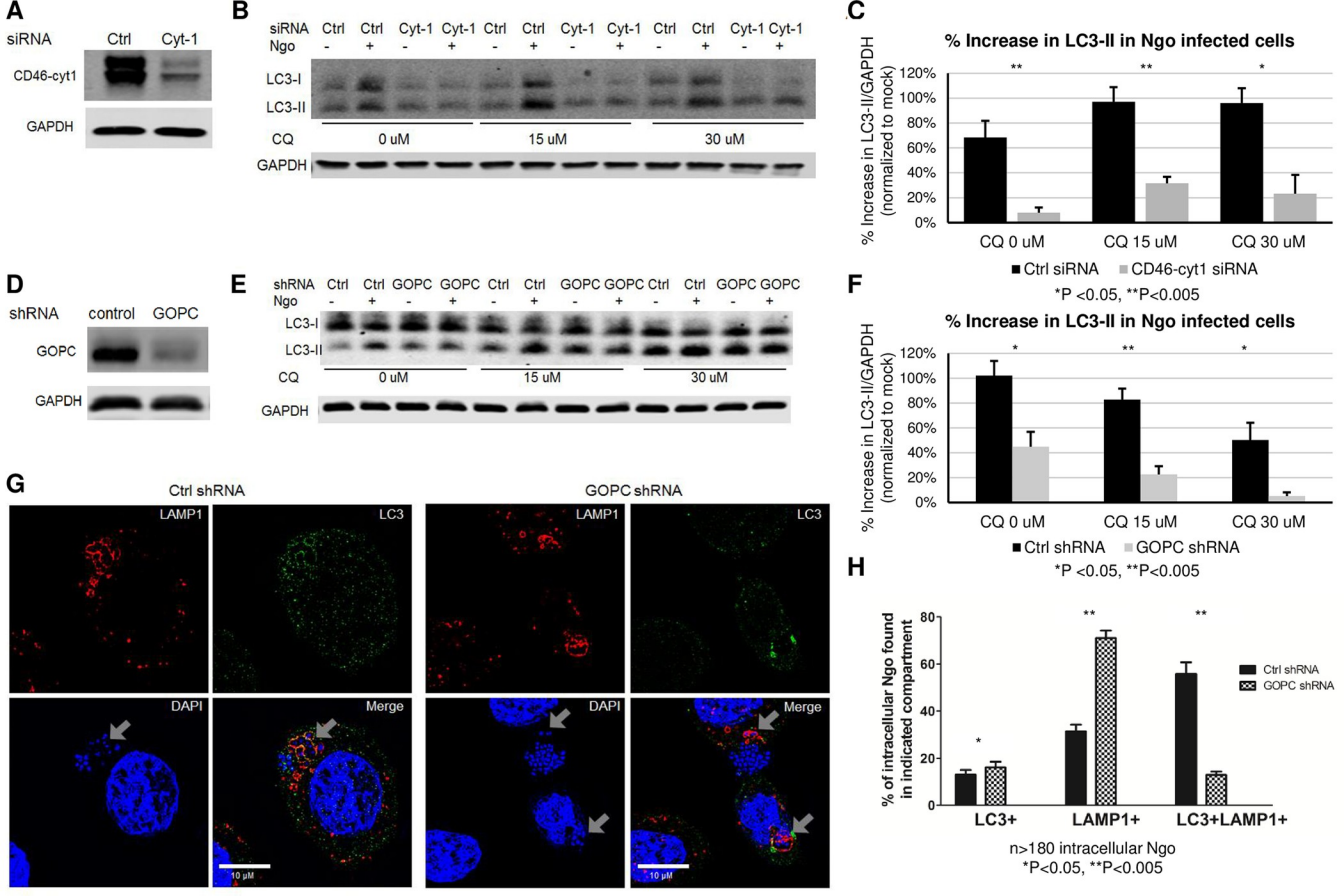
Autophagic flux in Ngo infected cells is mediated by CD46-cyt1. (A)Representative immunoblot showing CD46-cyt1 and GAPDH in ME180 cells treated with control (Ctrl) or CD46-cyt1 (Cyt-1) siRNA. GAPDH in each sample was used as the internal control. (B)Representative immunoblot showing LC3-I, LC3-II and GAPDH in ME180 cells treated with Ctrl or CD46-cyt1 (Cyt-1) siRNA, followed by treatment with 0, 15 or 30 μM CQ, and mock-infected or infected with Ngo at an MOI of 10 for 4 h. (C)Densitometry quantification of immunoblots from 4 independent experiments as described in (B). For each condition, LC3-II/GAPDH levels in infected cells were normalized to LC3-II/GAPDH levels in mock-infected controls. (D)Representative immunoblot showing GOPC and GAPDH in cells transduced with lentivirus containing either control shRNA or GOPC shRNA. (E)Representative immunoblot showing LC3-I, LC3-II and GAPDH in cells transduced with lentivirus containing control (Ctrl) or GOPC shRNA and mock-infected or infected with Ngo for 4 h at an MOI of 10. (F)Densitometry quantification of immunoblots from 5 independent experiments as described in (E). For each condition, LC3-II/GAPDH levels in infected cells were normalized to LC3-II/GAPDH levels in mock infected controls. (G)Representative SIM microscopy image of an ME180 cell treated with Ctrl or GOPC shRNA, infected with Ngo for 4 h at an MOI of 10, and stained for LAMP1 (red), LC3 (green), and DAPI (blue). Arrowheads show intracellular Ngo in intracellular compartments. (H)Quantification of intracellular Ngo found in LC3+, LAMP1+ or LC3+LAMP1+ compartments 4 hpi, in cells treated with Ctrl or GOPC shRNA.

The autophagic response is induced intrinsically by nutrient starvation or cellular stress, and extrinsically by the engagement of pattern recognition receptors (PRR) and CD46-cyt1 [3, 5, 14, 30, 31]. As Ngo induces CD46-cyt1 clustering at the site of infection, we tested the hypothesis that the autophagic response in Ngo-infected cells is mediated by CD46-cyt1 [16]. We knocked down CD46-cyt1 in ME180 cells using CD46-cyt1 siRNA (Cyt-1), and examined LC3-II levels upon Ngo infection. Under these conditions, we achieved ~61% downregulation of CD46-cyt1 (Fig 3A). Cells treated with control siRNA (Ctrl) served as the negative control.

Cells treated with Ctrl or Cyt-1 were infected with Ngo and LC3-II levels were measured by immunoblotting. Various concentrations of CQ were used to block the lysosome-dependent degradation of LC3-II, in order to measure the total accumulation of LC3-II. The increase in LC3-II levels in response to infection was significantly lower in Cyt1-treated cells compared to Ctrl-treated cells (Fig 3B and 3C), suggesting that CD46-cyt1 engagement is necessary for autophagy induction. To ensure that the autophagic response to Ngo is not cell-line specific, we knocked down CD46-cyt1 in Hec1B endometrial cells and quantitated LC3-II levels in infected cells (S4A Fig). CD46-cyt1 knockdown also resulted in a defective autophagic response in Ngo infected cells (S4B and S4C Fig).

Ngo recruitment of CD46-cyt1 to the site of infection is mediated by Tfp retraction; Δ*pilT*, a retraction-deficient mutant, fails to recruit CD46-cyt1 [15, 18]. We determined whether Δ*pilT* induces autophagy. Δ*pilT-* infected ME180 cells had visibly lower levels of LC3-II than cells infected with wt Ngo (S5 Fig). This result further indicates that Ngo induces autophagy through CD46-cyt1.

CD46-cyt1 regulates autophagy by interacting with the scaffold protein GOPC, which subsequently recruits the autophagy initiation complex VPS34 and Beclin-1 [14]. We downregulated GOPC in ME180 cells with shRNA and determined whether this would affect Ngo induction of autophagy. Under our conditions, ~65% knockdown of GOPC was achieved (Fig 3D). The increase in LC3-II levels in response to infection was significantly lower in knockdown cells than control cells (Fig 3E and 3F). Taken together, these results indicate that the autophagic response to Ngo infection is initiated by CD46-cyt1/GOPC.

Knocking down CD46-cyt1 and GOPC did not completely abolish the autophagic response (Fig 3C and 3F). This could reflect the incomplete knockdown of CD46-cyt1 and GOPC or the involvement of other pathways. Nevertheless, our results indicate that CD46-cyt1 and GOPC play an important role in the autophagy response to Ngo-infection.

We next determined whether knocking down GOPC affects the distribution of Ngo in autophagosomes/autophagolysosomes. In cells treated with control shRNA, 56% of intracellular Ngo were LC3+, LAMP1+ (Fig 3G and 3H). By contrast, only 13% of intracellular Ngo colocalized with these markers in GOPC-downregulated cells (Fig 3G and 3H). Strikingly, 71% of intracellular Ngo colocalized only with LAMP1, the lysosome/endosome marker (Fig 3G and 3H), indicating GOPC is involved in targeting intracellular Ngo to autophagolysosomes. These results support the hypothesis that GOPC is involved in the autophagic response to Ngo infection. Moreover, they suggest that the CD46-cyt1/GOPC autophagic pathway plays an important role in directing invading Ngo to autophagolysosomes.

**Fig 4:**
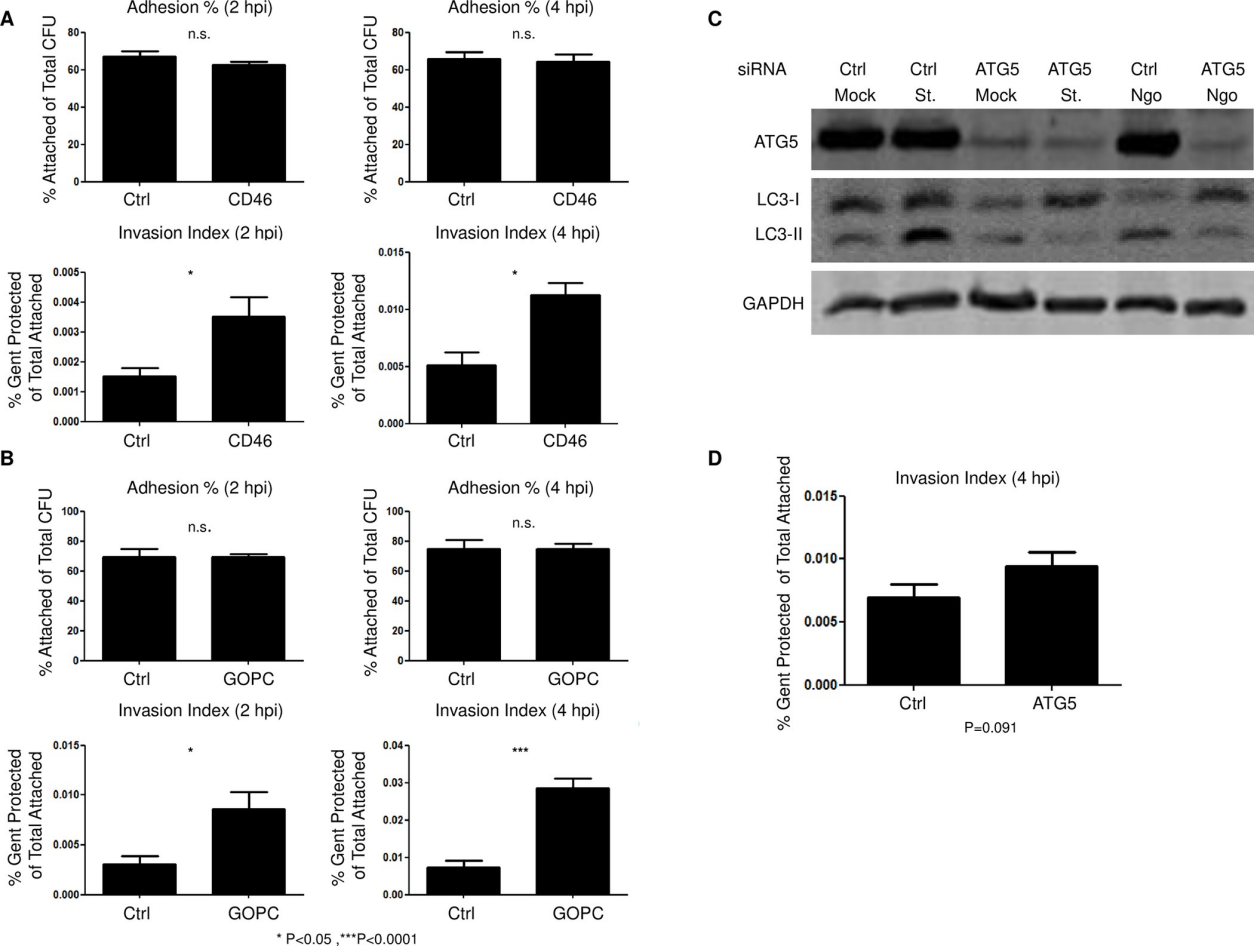
Downregulating CD46-cyt1 and GOPC reduces the viability of intracellular Ngo. (A)Attached Ngo and viable intracellular Ngo in ME180 cells treated with control (Ctrl) or CD46-cyt1 siRNA and infected at an MOI of 10, for 2 or 4 h. Attached CFUs were normalized to input CFUs (top panels); viable intracellular CFUs were normalized to attached CFUs (bottom panels). (n = 3). Error bars represent SEM. Statistical analysis was performed using student’s *t-*test. (B)Attached Ngo and viable intracellular Ngo in ME180 cells transduced with control (Ctrl) or GOPC lentiviral shRNA and infected at an MOI of 10, for 2 or 4 h. Attached CFUs were normalized to input CFUs (top panels); viable intracellular CFUs were normalized to attached CFUs (bottom panels) (n = 3–5). Error bars represent SEM. Statistical analysis was performed using student’s *t-*test. (C)Representative immunoblot showing ATG5, LC3-I, LC3-II and GAPDH levels in cells treated with control (Ctrl) or ATG5 siRNA, and mock infected, starved (St.) or infected with Ngo at an MOI of 10 for 4 h. (n = 5). (D)Viable intracellular Ngo recovered from ME180 cells treated with control (Ctrl) or ATG5 siRNA and infected at an MOI of 10 for 4 h. (n = 5). Error bars represent SEM. Statistical analysis was performed using student’s *t-*test.

We attempted to identify the membrane markers surrounding Ngo in CD46-cyt1-knockdown cells. Throughout infection, the vast majority of Ngo are on the cell surface; only a small percentage (~0.001–0.03%, Fig 4A and 4B) of attached MS11 survive inside cells, making it difficult to find intracellular bacteria [32]. Furthermore, we consistently recovered ~2-3-fold fewer intracellular Ngo in cells treated with siRNA transfection reagent (compare Invasion Index Fig 4A vs. 4B, and Figs 4A vs 5C). Thus, although we succeeded in visualizing intracellular Ngo in cells treated with Ctrl or CD46-cyt1 siRNA, we were unable to locate sufficient numbers to perform statistical analysis.

**Fig 5:**
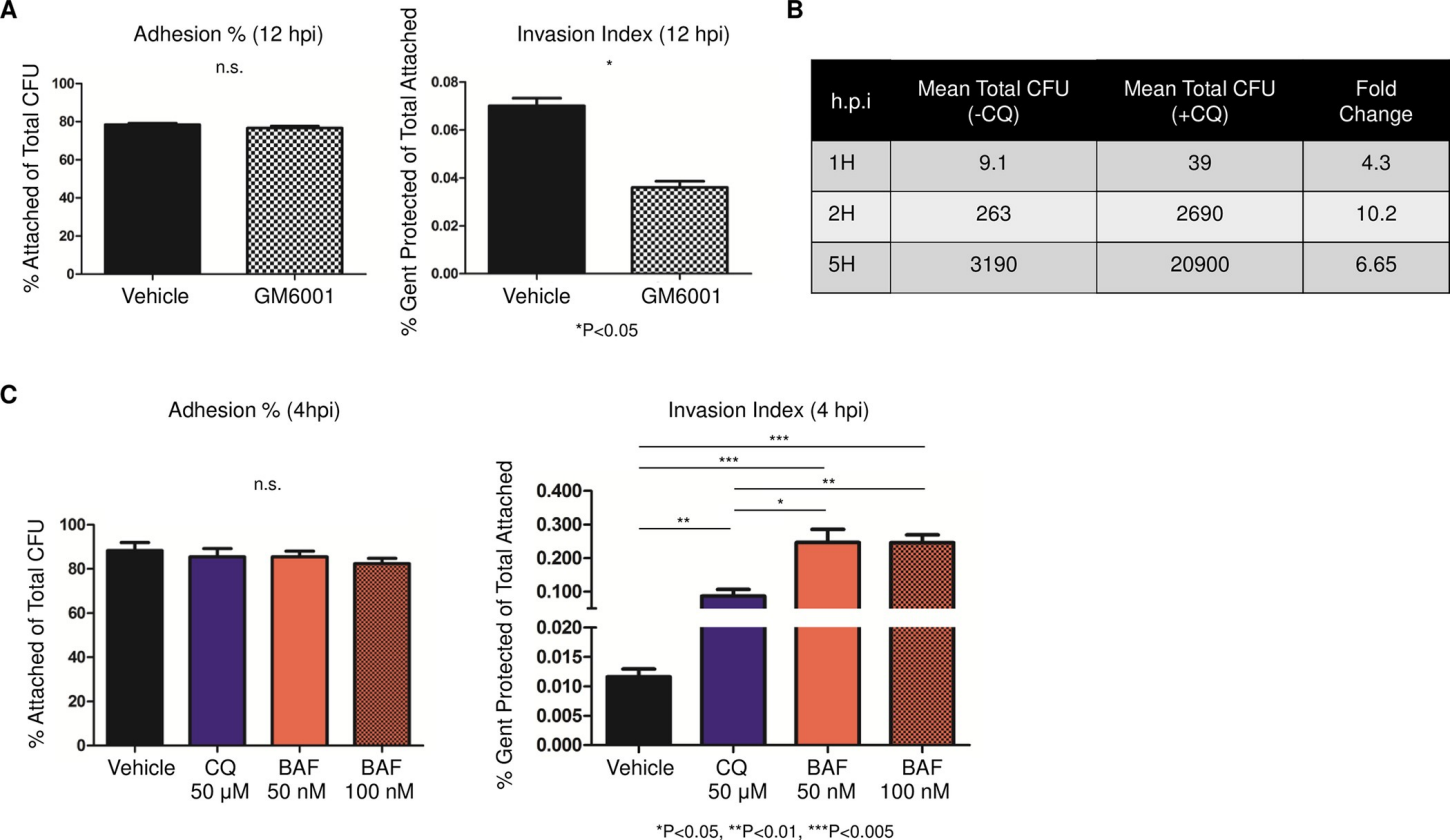
Lysosomal inhibitors increase the number of viable Ngo inside cells. (A)Attached and intracellular Ngo colony forming units (CFUs) in ME180 cells treated with GM6001 and infected at an MOI of 10, for 12 h. Attached CFUs were normalized to total CFUs (left); intracellular CFU was normalized to attached CFU (right) (n = 3). Error bars represent SEM. (B)Mean intracellular Ngo CFU in cells treated with chloroquine (+CQ) or vehicle (-CQ) at 1, 2, or 5 hpi (MOI = 10; n = 3). Statistical analysis was performed using student’s *t-*test. (C)Attached and intracellular Ngo CFUs in ME180 cells treated with CQ or Bafilomycin (BAF) for 4 h at the indicated concentration, at 4 hpi. Attached CFUs were normalized to total input CFUs (left); intracellular CFUs were normalized to attached CFUs (right) (n = 3). Error bars represent SEM. Statistical analysis was performed using student’s *t-*test.

### *Neisseria gonorrhoeae* is killed by the autophagic response

To determine whether the CD46-cyt1/GOPC autophagic response kills Ngo, CD46-cyt1 and GOPC knockdown cells were infected with Ngo for 2 or 4 h, and attached and intracellular bacteria were quantitated. Knocking down CD46-cyt1 did not affect Ngo attachment (Fig 4A, top panel). However, the knockdown cells yielded significantly more intracellular Ngo than those treated with control siRNA (Fig 4A, bottom panel). This increase could either be due to more bacteria invading cells or increased intracellular survival/growth. To distinguish between these possibilities, we determined whether CD46-cyt1 knockdown affects the total of number of Ngo entering cells. ME180 cells were infected with CFSE-labelled Ngo, the CFSE signal from the extracellular bacteria were quenched with Trypan Blue, and the levels of total intracellular Ngo were determined by flow cytometry [33]. Downregulating CD46-cyt1 did not affect the percentage of cells harboring intracellular Ngo or the average fluorescence intensity originating from intracellular bacteria (S6A and S6B Fig). Thus, blocking CD46-cyt1 expression does not affect bacterial invasion; rather, it increases Ngo intracellular survival/growth. Similarly, inhibiting autophagy via GOPC knockdown increased recovery of viable intracellular Ngo without affecting attachment to cells (Fig 4B). Taken together, these data strongly suggest that CD46-cyt1/GOPC mediated autophagy kills Ngo at 2–4 hpi.

CD46-cyt1/GOPC is one of several pathways that induce autophagy. For example, starvation-induced autophagy does not involve CD46-cyt1/GOPC [14]. We asked whether knocking down ATG5, a key component in both canonical and noncanonical autophagic pathways would affect Ngo intracellular survival. As expected, ATG5-downregulated cells failed to accumulate LC3-II during starvation or Ngo infection (Fig 4C). ATG5-downregulated cells yielded higher levels of intracellular Ngo, but this increase was not statistically significant (P = 0.091) (Fig 4D).

### Inhibiting CD46 ectodomain cleavage increases the viability of intracellular Ngo

In previous studies, we showed that Ngo induces the cleavage of the CD46-cyt1 ectodomain by unknown metalloproteinase(s) and its subsequent shedding [17]. This enables the cleavage of its transmembrane domain by Presenilin/γSecretase and the release of its cytoplasmic domain [17]. CD46-cyt1 downregulation occurs gradually; by 9 hpi, cellular levels of CD46-cyt1 are significantly reduced [15, 17]. The importance of CD46-cyt1 downregulation to Ngo infection is not understood. To test the hypothesis that this downregulation serves to counteract the induction of autophagy, we blocked the proteolytic cleavage of CD46-cyt1 using the broad-spectrum metalloproteinase inhibitor GM6001, and determined intracellular Ngo counts [17]. Consistent with this hypothesis, GM6001 treatment reduced the number of viable intracellular Ngo (Fig 5A).

### Inhibiting lysosome function increases Ngo intracellular survival

Earlier, we reported that Ngo remodels lysosomes by secreting IgAP, a protein that cleaves the major lysosomal membrane protein LAMP1 as well as human IgA [34, 35]. IgAP cleavage of LAMP1 is also a gradual process, but by 9 hpi infected A431 endocervical epithelial cells have dramatically reduced levels of LAMP1 and two other lysosomal markers that are not IgAP substrates [35–37].

We tested the hypothesis that this interference with lysosome homeostasis allows Ngo to eventually survive inside cells. ME180 cells were infected with Ngo for 1, 2 and 5 h in the presence or absence of CQ, and the numbers of viable intracellular bacteria were quantitated. CQ treatment increased Ngo intracellular yield by 4–10 fold (Fig 5B). Treating cells with Bafilomycin, another lysosome inhibitor, had a similar effect, increasing viable intracellular Ngo counts by 10–20 fold (Fig 5C). Similar results were observed in human primary endocervical epithelial cells: CQ or Bafilomycin significantly increased the recovery of intracellular Ngo at 4 hpi (S7 Fig). Neither CQ nor Bafilomycin affected Ngo attachment in ME180s or primary cells, suggesting that the increased yield of intracellular bacteria is due to an increase in intracellular survival. The recovery of intracellular Ngo from CQ-treated cells at 1 hpi shows the bacterium is able to invade quickly, but early invaders do not survive lysosome killing. Taken together, these results strongly suggest that Ngo promotes its intracellular survival by modulating autophagic components CD46-cyt1 and lysosomes.

## Discussion

Autophagy is a well-established host defense mechanism against intracellular pathogens. Cells mount an autophagic response against Group A *Streptococci* and measles virus via the CD46-cyt1/GOPC pathway, causing their clearance [14]. Cells target *Salmonella-*containing vacuoles for autophagic degradation in a NDP52-, optineurin-, and p62-dependent manner [10–13]. Conversely, intracellular pathogens have evolved means to counteract autophagic killing. For instance, *Legionella* evades autophagy using its effectors RavZ and Lpg1137 to cleave LC3 and Syntaxin17, respectively [38, 39]. *Shigella flexneri* employs VirA and IcsB to inactivate Rab1 and inhibit Atg5, respectively, to avoid being targeted to autophagosomes [40, 41]. For many pathogens, the mechanisms they use to evade autophagic killing are little understood.

In this report, we showed that *Neisseria gonorrhoeae* activates autophagy in primary human endocervical epithelial cells and two established endocervical cell lines. Autophagy is induced through the CD46-cyt1/GOPC pathway, intracellular Ngo are located in autophagosomes, and Ngo invading cells early in infection are killed.

**Fig 6:**
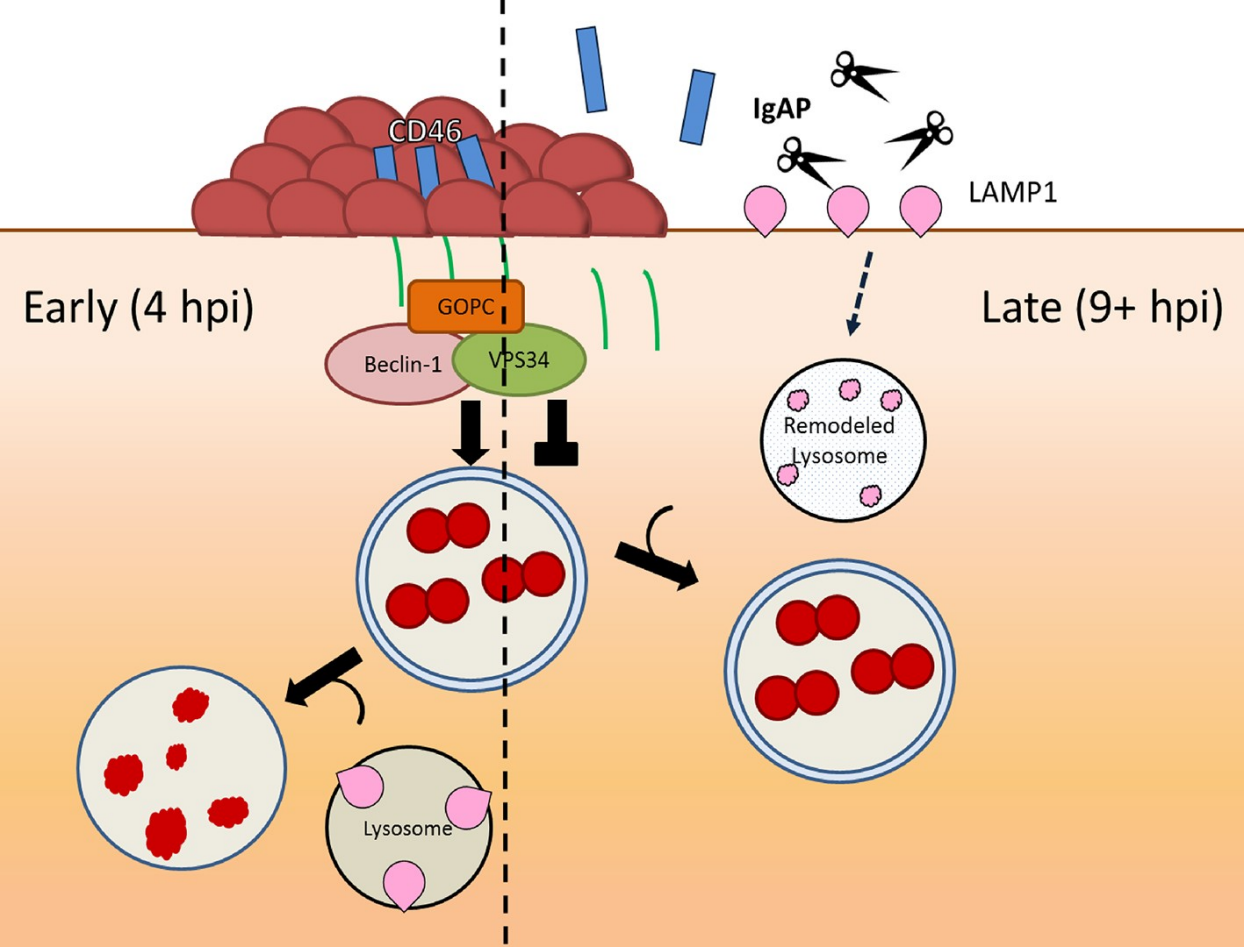
The duality of *N*. *gonorrhoeae*-epithelial interactions and how these interactions allow the pathogen to survive autophagic killing. Ngo adheres to and invades epithelial cells shortly after infection via its Tfp. Tfp retraction induces autophagy through the CD46-cyt1/GOPC pathway, reducing the number of viable bacteria invading cells at early stages of infection (left arrow). Throughout infection, Tfp retraction causes the shedding of the CD46-cyt1 ectodomain (blue rectangle) and the release of its cytoplasmic tail (green line), gradually reducing the intracellular pool of CD46-cyt1 and diminishing the ability of infected cells to initiate autophagy. Concurrently, the secreted IgAP cleaves LAMP1, ultimately remodeling lysosomes and blocking lysosome/autophagosome fusion and/or preventing degradation of autophagolysosomal contents (right arrow). This dual interference of the autophagic pathway promotes survival of Ngo invading at later stages of infection.

Later in infection, however, Ngo is able to survive inside cells. Ngo is known to slowly downregulate CD46-cyt1 [17] and disturb lysosome homeostasis [35–37]. We presented evidence that this dual interference ultimately counteracts the autophagic response to promote survival of late invaders (Fig 6, right section). Ngo is assumed to be weakly invasive because few viable intracellular bacteria are recovered early in infection. Our findings indicate Ngo has the ability to invade cells early, but these early invaders are killed by the autophagic response.

Recently, Ngo was reported to induce autophagy in HeLa cells, and this response limits the viability of the pathogen [42]. Whether CD46-cyt1/GOPC was involved in this autophagic response was not determined. Nevertheless, when both studies are taken into account, it is likely that the epithelial cell mounts an autophagic response to Ngo infection that kills early invaders. The above study also demonstrated that at a later stage of infection, i.e. 6 hpi, Ngo inhibits maturation of autophagosomes. Our results suggest that degradation of CD46-cyt1 and LAMP1 may be involved in this inhibition. However, it remains to be determined whether Ngo can accelerate CD46-cyt1 and LAMP1 degradation at high MOI condition (100) used in HeLa cell infection.

Ironically, the autophagic response mounted against early invaders may serve to benefit intracellular growth of late invaders. To thrive inside epithelial cells, Ngo must acquire iron from host sources [43]. To achieve this, Ngo disturbs iron homeostasis in the cell, inducing ferritin storage compartments to release bioavailable iron for its use [44–47]. As autophagy mediates ferritin degradation, the autophagic flux initiated early in infection may increase bioiron in the cell, promoting the growth of Ngo invading cells at later stages of the infection [48–50].

Finally, several Ngo surface proteins promote pathogen interactions with the epithelial cell, among them, the phase-variable Type IV pilus (Tfp) and Opa family of proteins [19, 51–58]. In our experiments, we used a piliated strain with its *opas* phase switched OFF, to avoid confounding effects stemming from Opa-mediated interactions. Our findings therefore specifically address autophagy in this strain background. In real life, Ngo cells recovered from an infected site are a mixed population, with surface proteins variously phase switched ON or OFF [59, 60]. It is tempting to speculate that if nonpiliated cells expressing an invasion-promoting Opa [51, 52, 56] predominate in the population that is being transmitted, then in the newly infected individual Ngo would invade cells through a different pathway, autophagy may not be induced, and Ngo might establish an early foothold inside the cell. Each Opa variant interacts with specific members of the carcinoembryonic antigen cell adhesion molecule (CEACAM). To date, CEACAMs have not been directly implicated in autophagy. However, Ngo invading cells through Opa are likely to interact with various intracellular TLRs and NLRs that regulate autophagy [31, 61–64]. Future work will determine whether and how phase variation of Ngo surface proteins affects autophagy and the lifecycle of the pathogen.

## Methods

### Reagents

Rabbit polyclonal LC3B (2775S), GOPC (8576S), and Rubicon (D9F7) antibodies were purchased from Cell Signaling Technology (Beverly, Massachusetts, USA). Mouse monoclonal GAPDH antibody was purchased from Thermo Fisher (Waltham Massachusetts, USA). Mouse monoclonal LAMP1 and CD46-cyt1 were generated in the lab. Alexa Fluor secondary goat polyclonal anti-rabbit and anti-mouse antibodies were purchased from Thermo Fisher. AG1478, an EGFR kinase inhibitor, was purchased from Calbiochem (San Diego, CA, USA). Transfection reagents siRNAmax and TurboFect, purchased from Invitrogen (Carlsbad, California, USA), were used according to manufacturer’s instructions. CD46-cyt1 siRNA (AUACCUAACUGAUGAGACCUU) was purchased from Dharmacon (Lafayette, Colorado, USA). Control and GOPC shRNA vectors pLKO.1 and A3-pLKO.1 were kindly provided by Dr. Jean Wilson (University of Arizona).

### Cell lines and RNAi

ME180 human endocervical epithelial cells (American Type Culture Collection, Manassas, Virginia, USA) and Hec1B human endometrium (ATCC) were maintained in McCoy’s (Gibco, Gaithersburg, Maryland, USA) and RPMI (Gibco) medium, respectively, containing 10% heat-inactivated filter-sterilized fetal bovine serum (FBS, Atlanta Biologicals, Flowery Branch, Georgia, USA) at 37°C and 5% CO_2_. Primary human cervical cells were a kind gift from Dr. McBride (NIAID, NIH). The primary cells were grown as previously described (pmid 29162712). Briefly, Cells were expanded in Rheinwald-Green F medium (3:1 Ham’s F-12/high-glucose Dulbecco’s modified Eagle’s medium (DMEM) with 5% fetal bovine serum (FBS, Sigma, St. Louis, Missouri, USA), 0.4 μg/ml hydrocortisone, 8.4 ng/ml cholera toxin, 10 ng/ml epidermal growth factor, and 24 μg/ml adenine, 6 μg/ml insulin) on a layer of lethally irradiated J2-3T3 murine fibroblasts. For routine culturing, the cells were grown in ROCK inhibitor 10 μM Y-27632 (Chemdea, USA) as described (pmid 20516646). The ROCK inhibitor was removed before the experiment. For siRNA transfection, 40% confluent cells were incubated in serum-free Opti-MEM (Invitrogen) for 16 h. Following transfection, cells were maintained in complete McCoy’s medium for 24 h, before infection. For lentivirus construction, 70% confluent 293T cells in RPMI (Gibco) containing 10% FBS were transfected with pLKO.1 or A3-pLKO.1, together with packaging and envelope plasmids psPAX2 and pMD2. 24 h after transfection, the medium was replaced with RPMI containing 30% FBS for 24 h. Supernatants were filtered through a 0.45 μm membrane to collect the lentivirus. 500 μL of filtered supernatant was added to 70% confluent ME180 cells for 24 h. Non-transduced cells were counterselected by incubating the culture with Puromycin (Sigma) (1.25 μg/mL) for 48 h. Transduced cells were frozen in liquid nitrogen (-180°C). The culture was immunoblotted to verify knock-down of target.

### Bacterial strains and infections

*Neisseria gonorrhoeae* (Ngo) strain MS11 was used for all infections and was maintained on GCB agar plus Kellogg’s supplements I and II at 37°C and 5% CO_2_. Only piliated and Opa-non expressing bacteria, as monitored by colony morphology, were used. For attachment and invasion experiments, bacteria resuspended in GCB liquid medium were added to epithelial cells at a multiplicity of infection (MOI) of 10, unless otherwise stated. Infections were performed in 12-well plates (Falcon, Corning, New York, USA). To determine the viable intracellular CFU, cells were treated gentamicin (50 μg/mL) for 1 h at 37°C to kill extracellular bacteria. Cells were then washed three times with liquid GCB and treated with GCB containing 0.5% (wt/vol) saponin (Sigma) for 15 minutes. Serial dilutions of cells scraped with 1 mL pipette were plated for intracellular count.

### Immunoblotting

ME180 cells were mock-infected with GCB medium alone or with Ngo, for the indicated times. Starvation, as a positive control for induction of autophagy, was performed by incubating cells in Earle’s Balanced Salt Solution (Gibco). To induce LC3-II accumulation, cells were incubated with chloroquine diphosphate (Sigma) or bafilomycin A1 (Sigma) for 1 h prior to infection and maintained in the medium throughout the experiment. To terminate infection, unattached bacteria were removed by washing the cultures twice with ice-cold PBS. Cells were then lysed with 120 μL of RIPA2 lysis buffer (150 mM NaCl, 5 mM EDTA pH 8.0, 50 mM Tris pH 8.0, 1.0% NP-40, 0.5% sodium deoxycholate, 0.1% SDS). Lysates were mixed 1:1 with Tris-Tricine sample buffer (Bio-Rad, Hercules, California, USA) and 1 tablet of protease inhibitor cocktail (Roche, Indianapolis, Indiana, USA). The samples were boiled for 10 min, and then separated in a SDS 12% Tris-Tricine polyacrylamide gel containing urea (6M). The separated proteins were transferred to PVDF membranes (0.1 μm, GE, Fairfield, Connecticut, USA) and probed with the appropriate antibodies (overnight at 4°C).

### Immunofluorescence microscopy

Cells were grown on #1.5 thickness coverslips (Zeiss, Thornwood, New York, USA) coated with fibronectin (Sigma), to 50–60% confluency. Mock- or Ngo-infected cells were washed with room temperature PBS 3X and fixed with methanol-free 4% paraformaldehyde for 20 min room temperature. Cells were blocked with PBS containing normal goat serum (3%, w/v) and saponin (0.03%, w/v). Primary antibodies were used at the following dilutions: 1:20 for staining Ngo, 1:40 for LAMP1, 1:100 for LC3 and Rab5, and staining was allowed to proceed at 4°C overnight. Staining with secondary antibodies was performed at a 1:1000 dilution for 1 h at room temperature. Samples were mounted with 20 μL of Pro-long Gold (Thermo Fisher). The mounting medium was allowed dry for 24 h for Deltavision (GE, Lifesciences North Imaging Cores) Microscopy and 120 h for Zeiss Structure-Illumination Microscopy. Images were analyzed on Zen Black software (Zeiss).

### Quantification of intracellular Ngo using flow cytometry

Detailed protocol for flow cytometry based quantification of intracellular Ngo has been reported previously [33]. Briefly, MS11 (1x10^9^ CFU in 1 mL) was washed twice with PBS and suspended in 1 mL of PBS containing 1 μg of CFSE (Molecular Probes, Eugene, Oregon, USA). The bacteria were incubated for 25 min at 37°C with constant shaking, and washed three times with RT PBS. ME180 cells in 12-well plates were infected with labeled MS11 for 4 h. Following infection, cells were washed x2 with RT PBS and treated with 200 μL of trypsin at 37°C for 10 minutes. Detached cells resuspended and washed x2 in ice cold FACS buffer (PBS + 5% FBS). For quenching of extracellular CFSE signal, Trypan Blue (Sigma) was added to final concentration of 0.4%. FlowJo V10 software was used for data analysis.

### Statistics

Statistical analysis was performed using standard student *t-test* analysis with GraphPad 5.0 (San Diego, California, USA).

## Supporting information

S1 Fig Ngo induces LC3-II in the presence of lysosomal inhibitor. Densitometry quantification of immunoblots in Fig 1G (n = 2). As described, ME180 cells were mock infected, starved (St.), or infected with Ngo for 4 h in the presence of indicated concentrations of CQ. LC3-II levels normalized to internal control GAPDH were compared to those of mock infection.
(TIF)

S2 Fig Rubicon expression is undetectable in ME180s. (A) Representative immunoblot showing Rubicon and GAPDH in ME180 cells and Bone Marrow Derived Macrophages (BMDM). GAPDH in each sample was used as the internal control.
(B) Densitometry quantification of immunoblots from 2 independent experiments described in (A). Rubicon levels in uninfected ME180s and BMDMs were normalized to the internal control GAPDH.
(TIF)

S3 Fig Intracellular Ngo colocalize with autophagolysosomal markers (LC3+LAMP1+) throughout the length of the cell. Successive SIM Z-sections of a field of Ngo-infected ME180 cells. LAMP1, LC3, and DAPI are red, green, and blue, respectively. Bot: bottom-most Z section. Top: Top-most Z seection. Most intracellular Ngo colocalized with LAMP1+, LC3+ compartments (autophagolysosomes) throughout the length of the cell.
(TIF)

S4 Fig Ngo infection induces autophagic flux in human endocervical Hec1B epithelial cells via CD46-cyt1. (A) Representative immunoblot showing CD46-cyt1 and GAPDH in cells treated with control (Ctrl) or CD46-cyt1 (Cyt-1) siRNA. GAPDH in each sample was used as the internal control.
(B) Representative immunoblot showing LC3-I, LC3-II and GAPDH in cells treated with Ctrl or Cyt-1 siRNA. Cells were treated with 0, 15 or 30 uM CQ, and mock infected or infected with Ngo at an MOI of 10 for 4 h.
(C) Densitometry quantification of immunoblots from 3 independent experiments as described in (B). LC3-II levels in Ngo infected cells were normalized to the GAPDH internal control, and compared to those from mock infected cells. Statistical analysis was performed using student’s *t-*test.
(TIF)

S5 Fig Autophagic flux in Ngo infected cells is mediated by Tfp retraction. (A) Representative immunoblot showing LC3-I, LC3-II and GAPDH in ME180 cells that were mock infected or infected with Ngo wt or Δ*pilT* at MOI of 10 for 4 h GAPDH served as the internal control for each sample.
(B) Densitometry quantification of LC3-II levels in immunoblots from 2 independent experiments described in (A). In each lane, the LC3-II signal was normalized to the GAPDH signal, and the normalized value was expressed relative to that in mock-infected cells.
(TIF)

S6 Fig CD46-cyt1 knockdown does not affect Ngo invasion. (A) Flow cytometry analysis of ME180 cells treated with control (Ctrl) or CD46-cyt1 (Cyt-1) siRNA and mock infected or infected with CFSE-labeled Ngo at an MOI of 10, for 4 h (n = 3). Prior to analysis, extracellular CFSE signal was quenched with Trypan Blue (final concentration 0.4%). Live population of cells was approximated using FSC-A vs. SSC-A plot (potential cell debris and dead cells with low FSC-A were removed from further analysis). Intracellular CFSE signals in live population were analyzed by CFSE histogram plots. The threshold for CFSE+ population was determined using mock infected cells (<0.01% cells in CFSE+ group). Identical gating schemes were applied to all experimental conditions.
(B) Quantification of the percentage of infected ME180 cells harboring intracellular Ngo (left) and CFSE mean fluorescence intensity of intracellular Ngo in CFSE+ population (right) (n = 3).
(TIF)

S7 Fig Lysosomal inhibitors increase the number of viable intracellular Ngo in human primary human endocervical epithelial cells. Quantitation of attached and intracellular Ngo colony forming units (CFU) in primary cells treated with CQ (50 μM) or Bafilomycin (50 nM) followed by infection at an MOI of 10 for 4 h. Attached CFUs were normalized to total input CFUs (left); intracellular CFUs were normalized to attached CFUs (right) (n = 3). Error bars represent SEM. Statistical analysis was performed using student’s *t-*test.
(TIF)

S1 Video LC3+, LAMP1+ signals surrounding intracellular Ngo resemble spherical/ellipsoidal vesicles. (A) Representative 3D reconstruction of SIM Z-sections (from bottom to top) of Ngo infected ME180 cells. LAMP1, LC3, and DAPI are red, green, and blue, respectively. Arrows indicate intracellular Ngo (cluster of DAPI signals in LC3+, LAMP1+, or LC3+LAMP1+ compartments).
(B) The infected cell shown in (A) was used to generate 3D reconstructions of LAMP1 and LC3 signals separately. LAMP1, LC3, and DAPI are red, green, and blue, respectively. Arrows indicate intracellular Ngo (cluster of DAPI signals in LC3+, LAMP1+ or LC3+LAMP1+ compartments).
(PPTX)
