# Supplementary figures and images for: Neisseria gonorrhoeae evades autophagic killing by downregulating CD46-cyt1 and remodeling lysosomes
Source: PLoS Pathog. 2019 Feb 12;15(2):e1007495. doi: 10.1371/journal.ppat.1007495 (PMC6388937; doi:10.1371/journal.ppat.1007495)

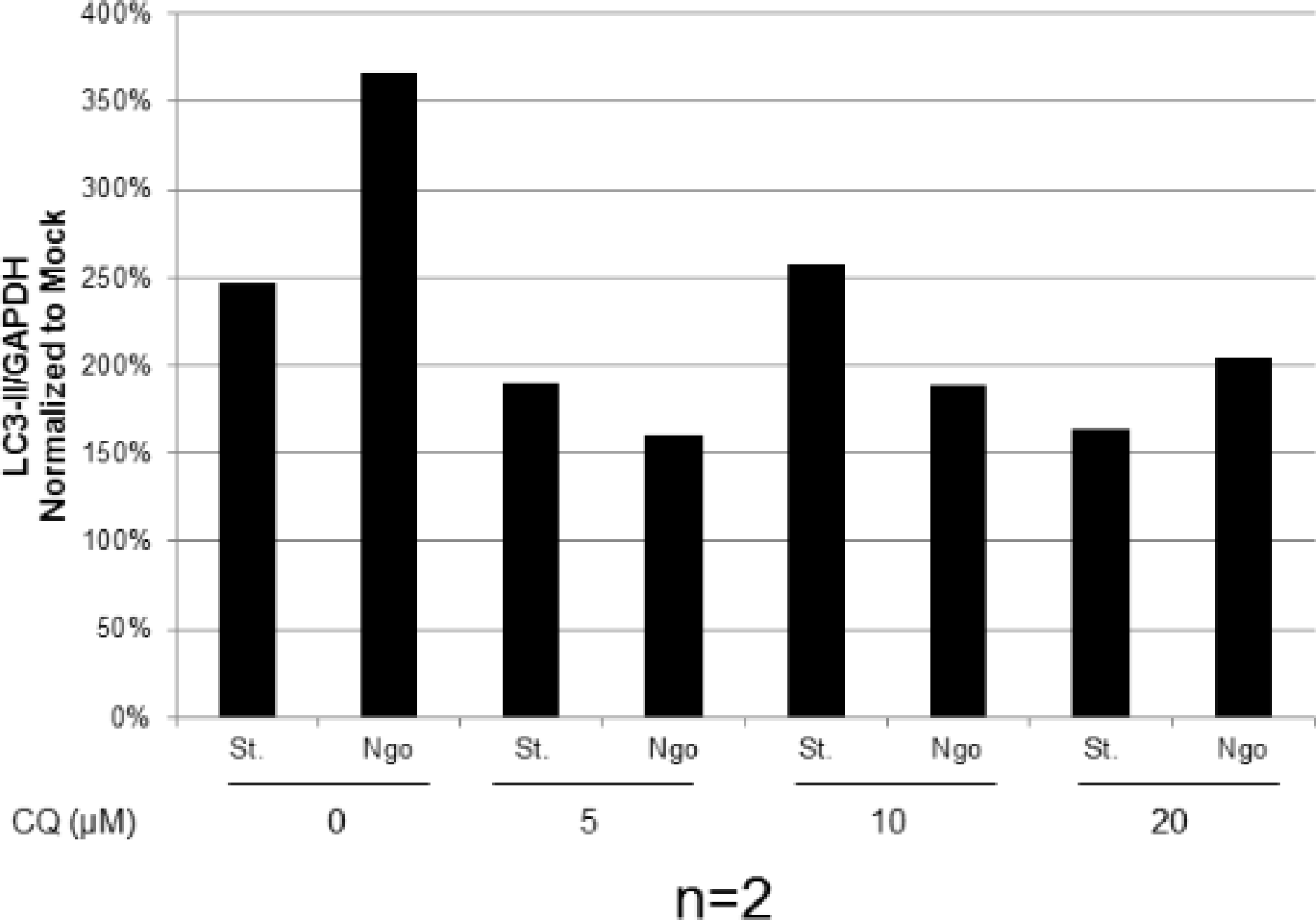

Supplement: S1 Fig — Densitometry quantification of immunoblots in Fig 1G (n = 2). As described, ME180 cells were mock infected, starved (St.), or infected with Ngo for 4 h in the presence of indicated concentrations of CQ. LC3-II levels normalized to internal control GAPDH were compared to those of mock infection. (TIF) [file ppat.1007495.s001.tif]

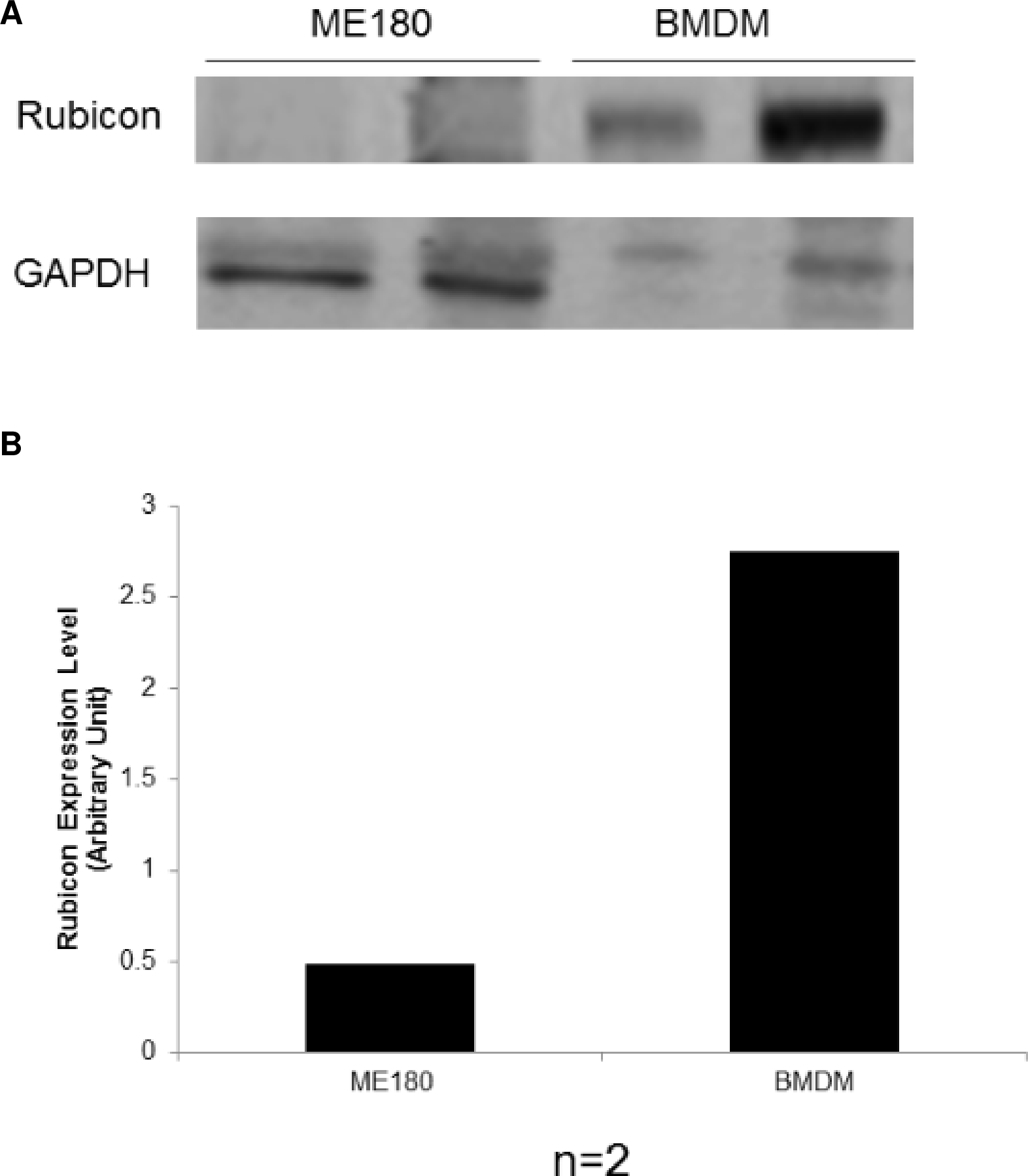

Supplement: S2 Fig — (A) Representative immunoblot showing Rubicon and GAPDH in ME180 cells and Bone Marrow Derived Macrophages (BMDM). GAPDH in each sample was used as the internal control. (B) Densitometry quantification of immunoblots from 2 independent experiments described in (A). Rubicon levels in uninfected ME180s and BMDMs were normalized to the internal control GAPDH. (TIF) [file ppat.1007495.s002.tif]

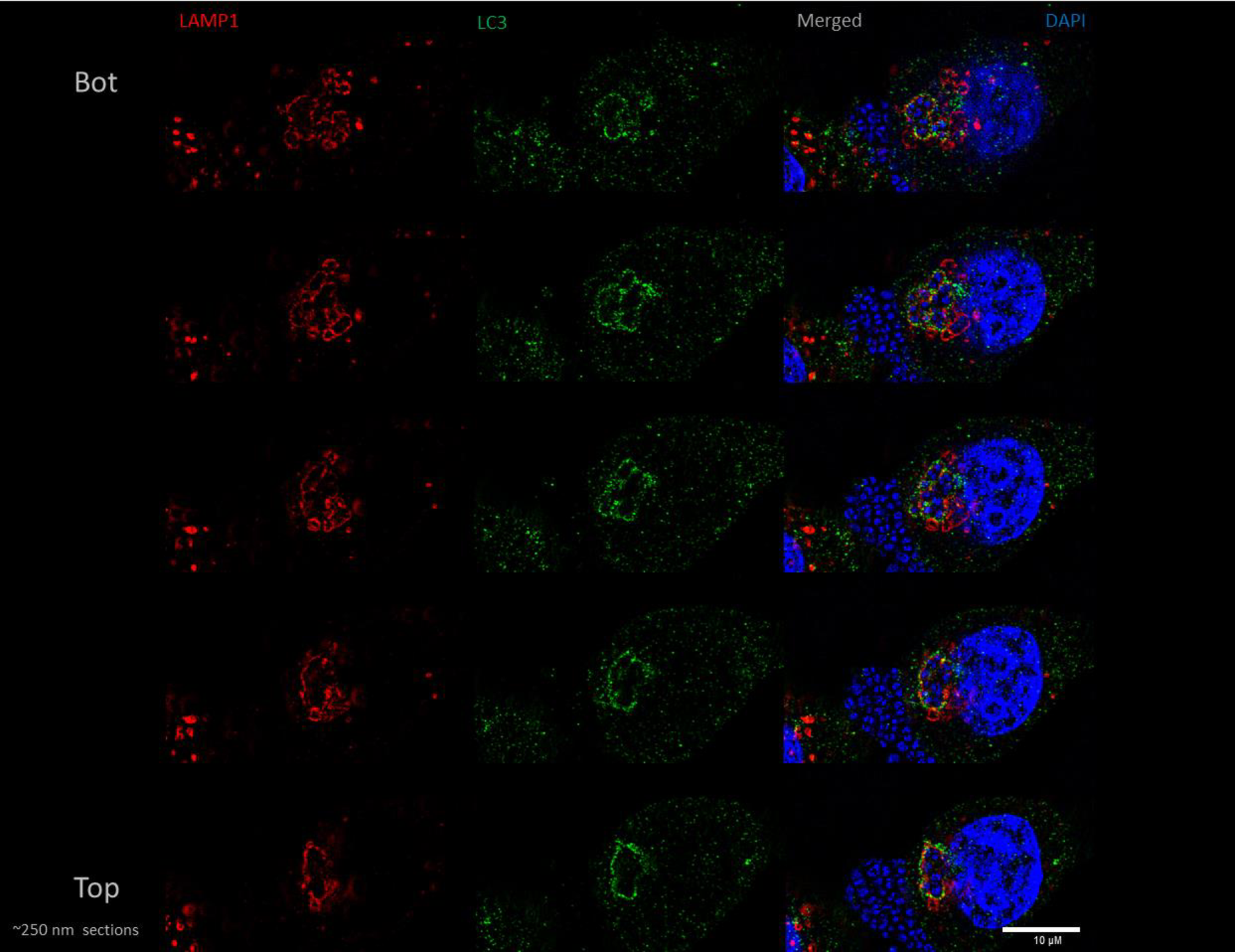

Supplement: S3 Fig — Successive SIM Z-sections of a field of Ngo-infected ME180 cells. LAMP1, LC3, and DAPI are red, green, and blue, respectively. Bot: bottom-most Z section. Top: Top-most Z seection. Most intracellular Ngo colocalized with LAMP1+, LC3+ compartments (autophagolysosomes) throughout the length of the cell. (TIF) [file ppat.1007495.s003.tif]

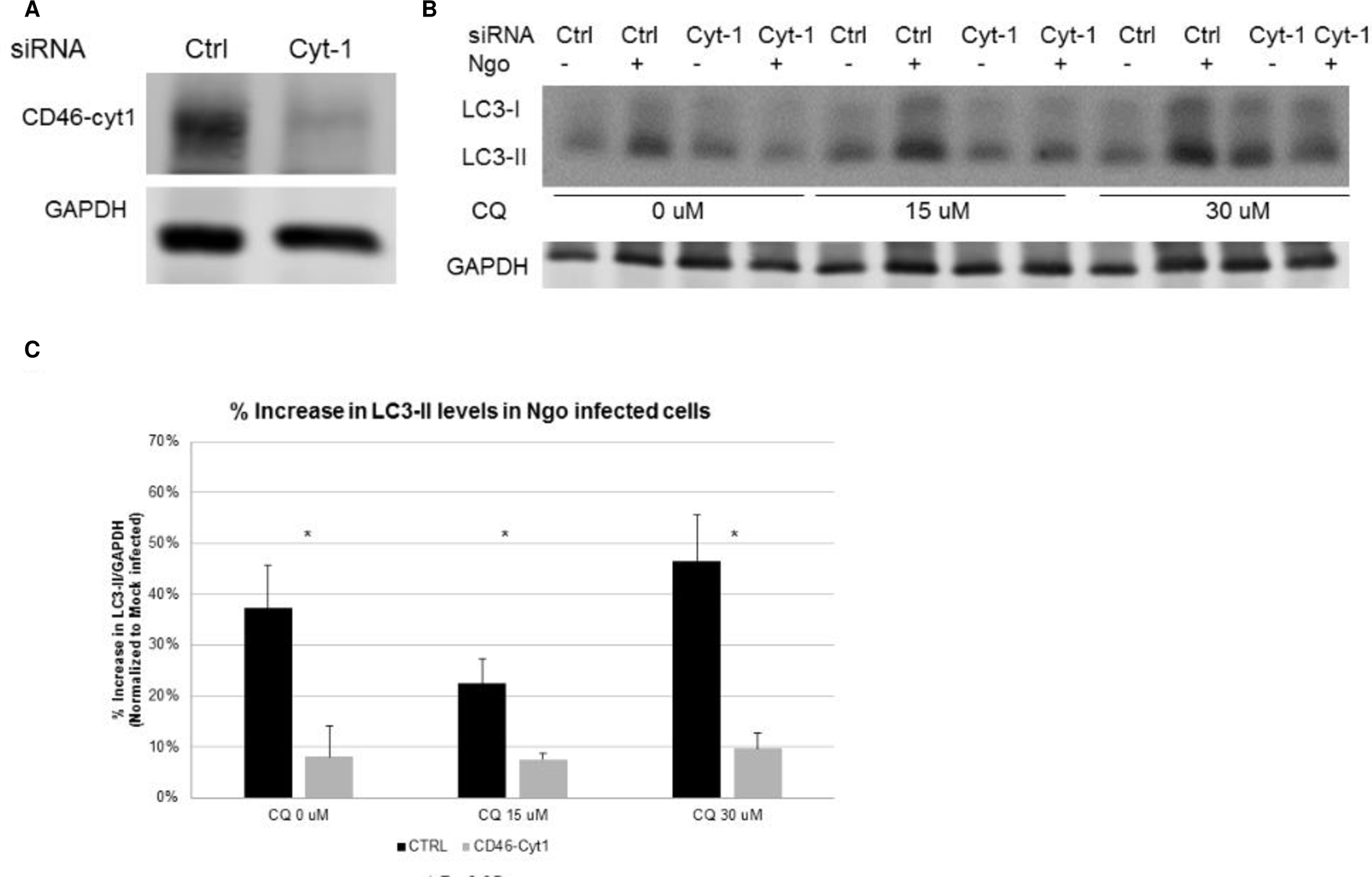

Supplement: S4 Fig — (A) Representative immunoblot showing CD46-cyt1 and GAPDH in cells treated with control (Ctrl) or CD46-cyt1 (Cyt-1) siRNA. GAPDH in each sample was used as the internal control. (B) Representative immunoblot showing LC3-I, LC3-II and GAPDH in cells treated with Ctrl or Cyt-1 siRNA. Cells were treated with 0, 15 or 30 uM CQ, and mock infected or infected with Ngo at an MOI of 10 for 4 h. (C) Densitometry quantification of immunoblots from 3 independent experiments as described in (B). LC3-II levels in Ngo infected cells were normalized to the GAPDH internal control, and compared to those from mock infected cells. Statistical analysis was performed using student’s t-test. (TIF) [file ppat.1007495.s004.tif]

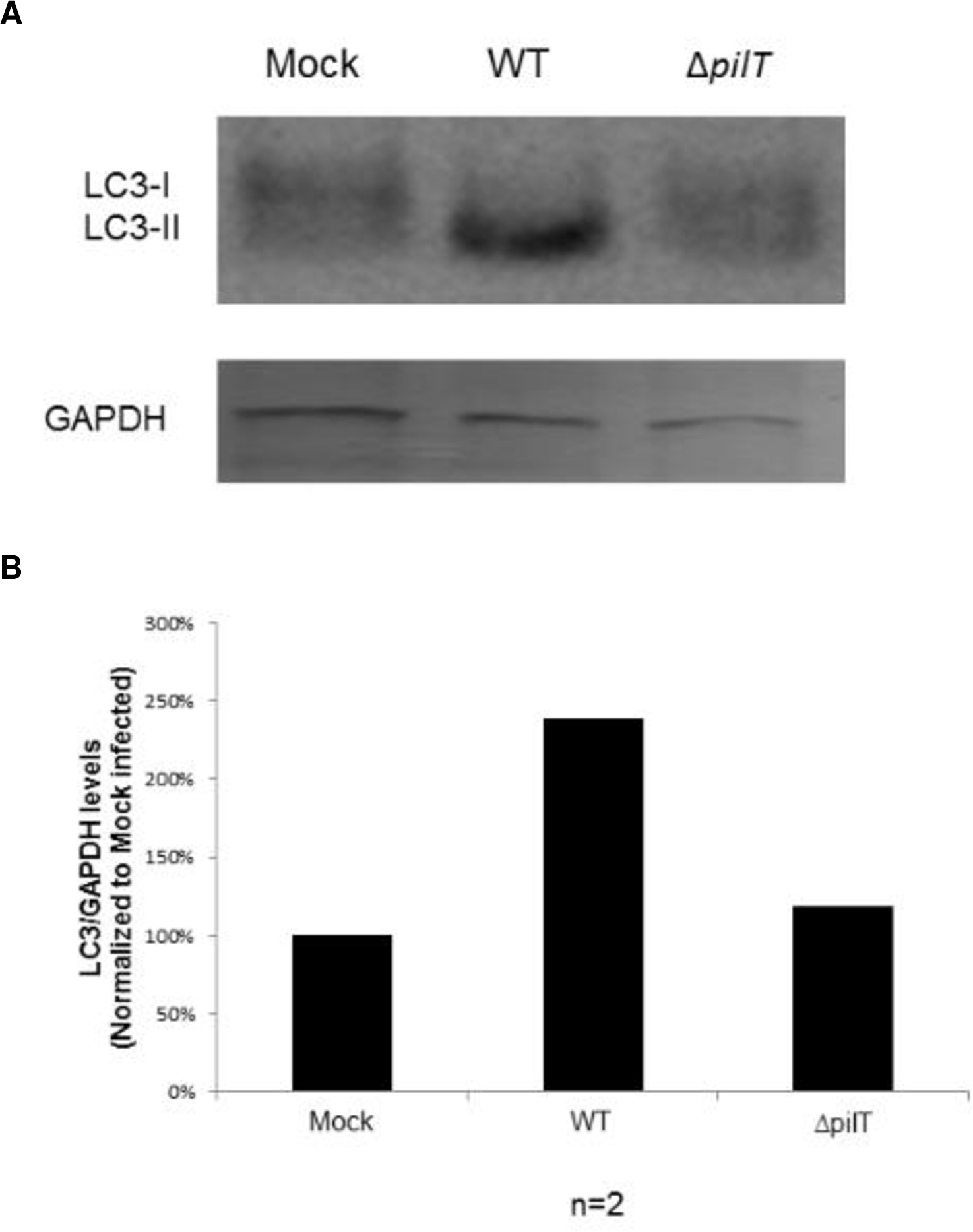

Supplement: S5 Fig — (A) Representative immunoblot showing LC3-I, LC3-II and GAPDH in ME180 cells that were mock infected or infected with Ngo wt or ΔpilT at MOI of 10 for 4 h GAPDH served as the internal control for each sample. (B) Densitometry quantification of LC3-II levels in immunoblots from 2 independent experiments described in (A). In each lane, the LC3-II signal was normalized to the GAPDH signal, and the normalized value was expressed relative to that in mock-infected cells. (TIF) [file ppat.1007495.s005.tif]

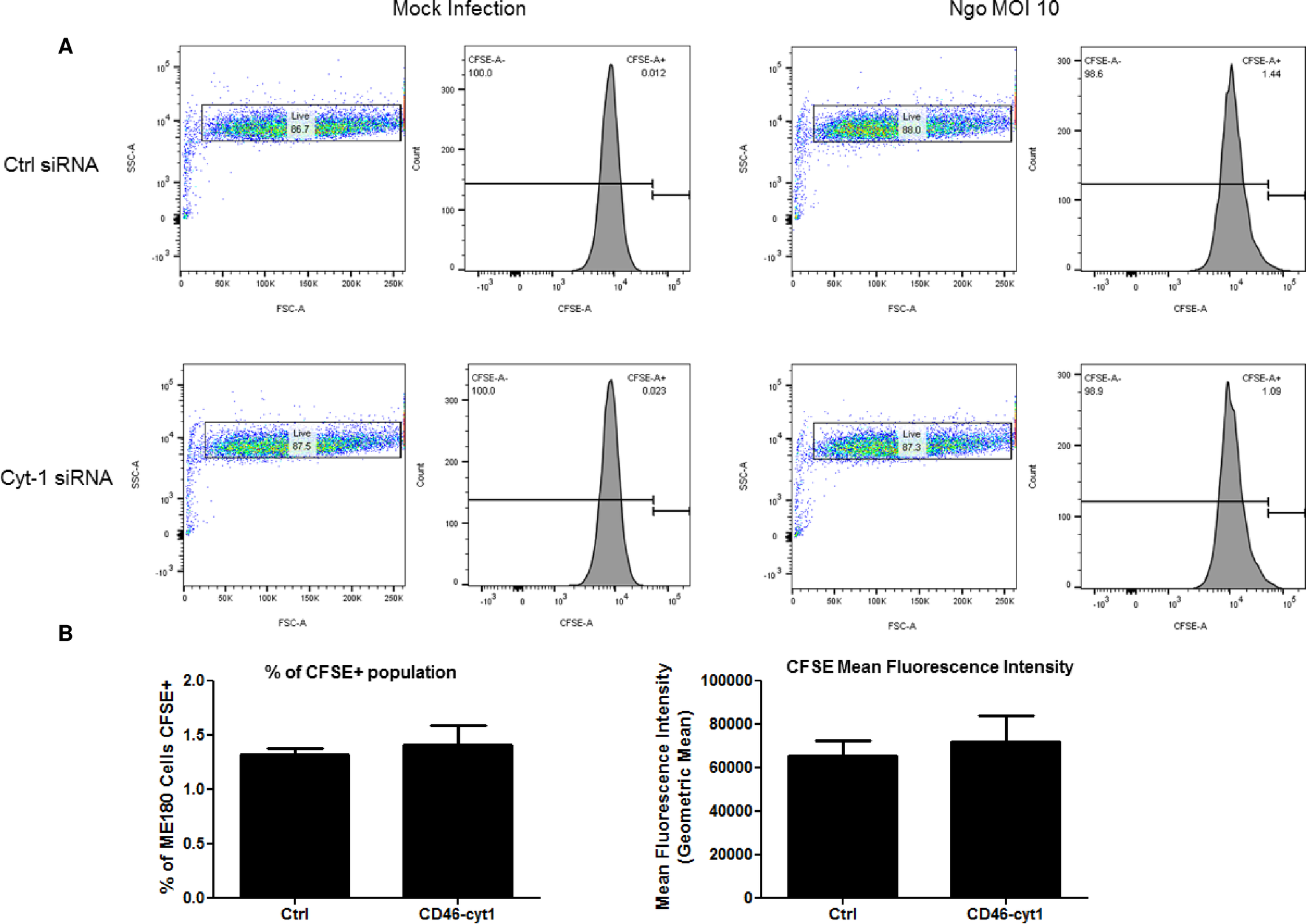

Supplement: S6 Fig — (A) Flow cytometry analysis of ME180 cells treated with control (Ctrl) or CD46-cyt1 (Cyt-1) siRNA and mock infected or infected with CFSE-labeled Ngo at an MOI of 10, for 4 h (n = 3). Prior to analysis, extracellular CFSE signal was quenched with Trypan Blue (final concentration 0.4%). Live population of cells was approximated using FSC-A vs. SSC-A plot (potential cell debris and dead cells with low FSC-A were removed from further analysis). Intracellular CFSE signals in live population were analyzed by CFSE histogram plots. The threshold for CFSE+ population was determined using mock infected cells (<0.01% cells in CFSE+ group). Identical gating schemes were applied to all experimental conditions. (B) Quantification of the percentage of infected ME180 cells harboring intracellular Ngo (left) and CFSE mean fluorescence intensity of intracellular Ngo in CFSE+ population (right) (n = 3). (TIF) [file ppat.1007495.s006.tif]

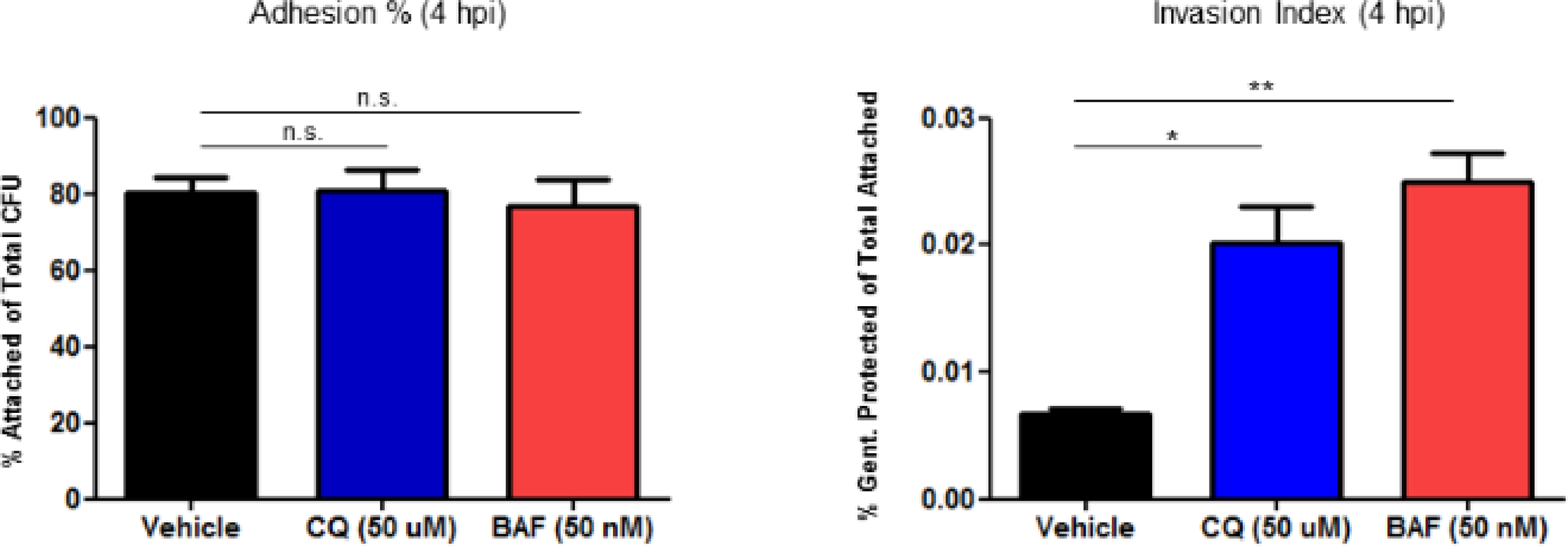

Supplement: S7 Fig — Quantitation of attached and intracellular Ngo colony forming units (CFU) in primary cells treated with CQ (50 μM) or Bafilomycin (50 nM) followed by infection at an MOI of 10 for 4 h. Attached CFUs were normalized to total input CFUs (left); intracellular CFUs were normalized to attached CFUs (right) (n = 3). Error bars represent SEM. Statistical analysis was performed using student’s t-test. (TIF) [file ppat.1007495.s007.tif]

## Slide 1
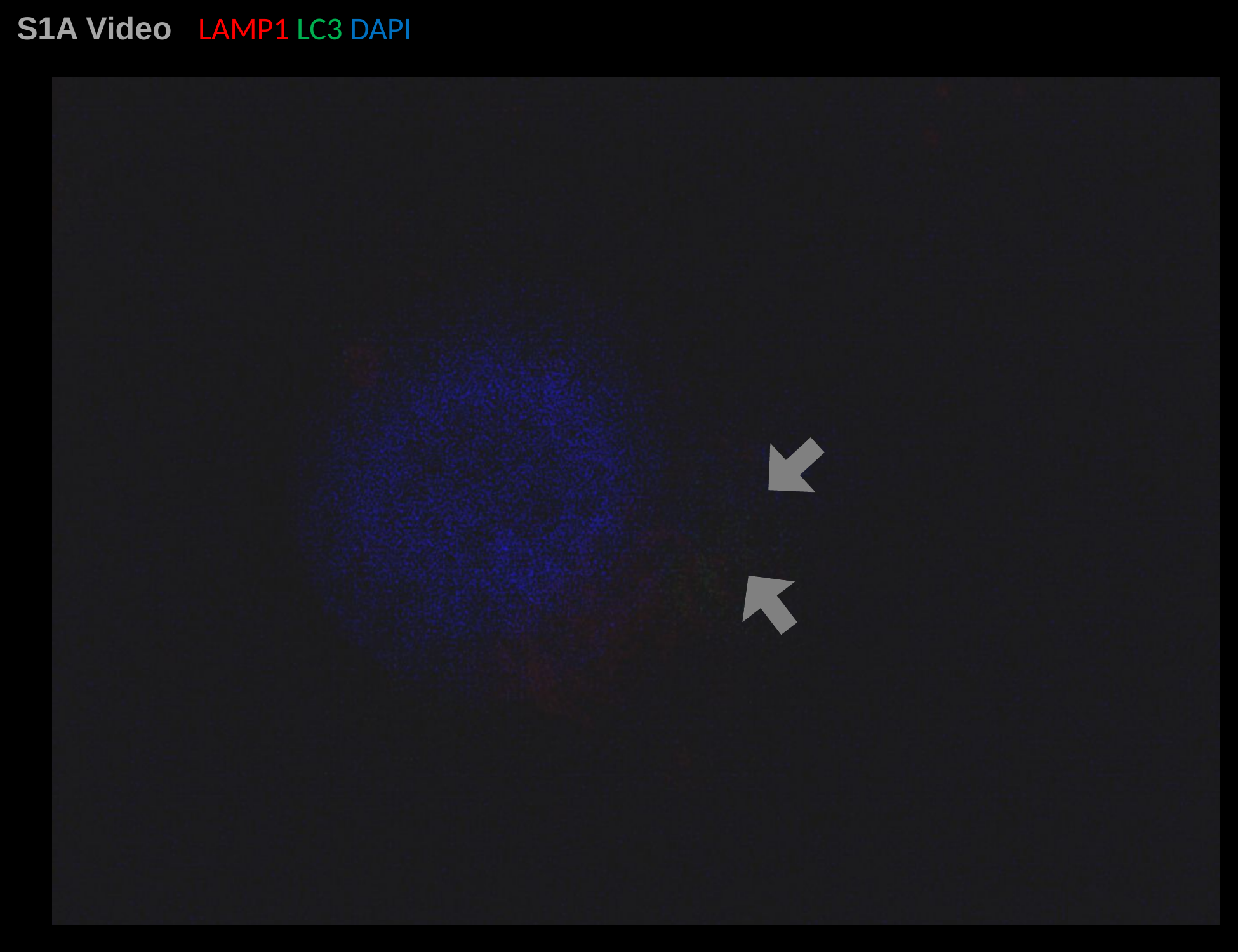

S1A Video
LAMP1 LC3 DAPI

## Slide 2
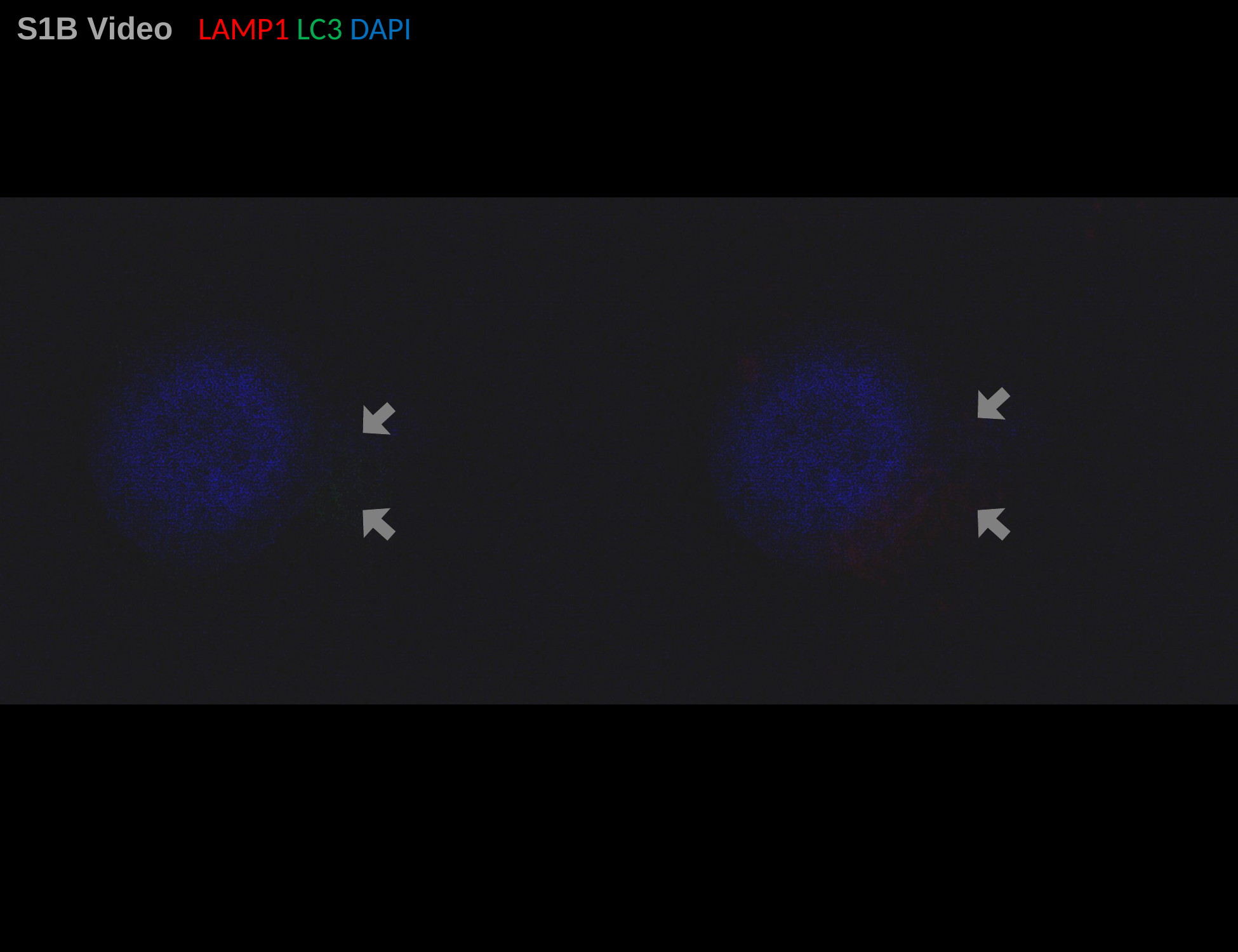

S1B Video
LAMP1 LC3 DAPI

Supplement: S1 Video — (A) Representative 3D reconstruction of SIM Z-sections (from bottom to top) of Ngo infected ME180 cells. LAMP1, LC3, and DAPI are red, green, and blue, respectively. Arrows indicate intracellular Ngo (cluster of DAPI signals in LC3+, LAMP1+, or LC3+LAMP1+ compartments). (B) The infected cell shown in (A) was used to generate 3D reconstructions of LAMP1 and LC3 signals separately. LAMP1, LC3, and DAPI are red, green, and blue, respectively. Arrows indicate intracellular Ngo (cluster of DAPI signals in LC3+, LAMP1+ or LC3+LAMP1+ compartments). (PPTX) [file ppat.1007495.s008.pptx]
